# Pepper root exudate attenuates snap bean root rot by mediating microbial community remodeling

**DOI:** 10.1128/aem.01664-25

**Published:** 2025-10-31

**Authors:** Ying Li, Le Liu, Huaiyuan Teng, Liqin Zhao, Bowen Fan, Fengjun Yang

**Affiliations:** 1 College of Horticulture and Landscape Architecture, Heilongjiang Bayi Agricultural University 91625, Daqing, China; The University of Tennessee Knoxville, Knoxville, Tennessee, USA

**Keywords:** root exudates, snap bean, microbial community, metabolites, root rot

## Abstract

Intercropping, as a sustainable agricultural practice, has shown potential to reduce soil-borne diseases through rhizosphere microbial community remodeling. However, whether pepper (*Capsicum annuum L*.) confers this benefit to snap bean (*Phaseolus vulgaris L*.) remains unclear. This study investigated the disease-suppressive effects of exogenously applied pepper root exudates using pot experiments. *In vitro* assays were used to validate the biocontrol efficacy of antagonistic bacteria and a key flavonoid metabolite (Chrysin). High-throughput sequencing and untargeted metabolomics were combined to analyze changes in the snap bean rhizosphere microbial community and root exudate profiles under pathogen stress. Compared to the treatment inoculated only with *Fusarium solani* (*F. solani*) (CKI), the treatment applying root exudates plus *F. solani* (REI) exhibited a substantial reduction in *F. solani* abundance, enhanced plant antioxidant enzyme activities, as well as markedly alleviated snap bean root rot symptoms. Furthermore, the REI treatment significantly altered rhizosphere microbial community composition by selectively enriching beneficial microbes (e.g., Actinobacteria, Ascomycota, *Streptomyces*, *Saccharothrix*), enhanced the complexity and stability of the bacterial-fungal cross-domain network, and formed a pathogen antagonism network centered on *Streptomyces*. Moreover, the REI treatment induced the secretion of flavone compounds (e.g., Chrysin) from snap bean roots, whose relative abundance demonstrated an essential positive correlation with *Streptomyces* and a significant negative correlation with *Fusarium*. The findings indicate that exogenous application of pepper root exudates mitigates the incidence of snap bean root rot by restructuring the rhizosphere microbial community and promoting the synthesis of endogenous flavone metabolites in the host plant. This study provides a basis for environmentally friendly vegetable disease management and supports the advancement of sustainable agricultural practices.

**IMPORTANCE:**

Root rot induced by *Fusarium solani* poses a serious threat to snap bean production and the sustainable development of agriculture. Long-term continuous cropping intensifies the incidence of soilborne diseases. Conventional chemical control methods are frequently less effective against these diseases and may result in issues, such as the development of pathogen resistance, a decline in soil microbial diversity, and environmental contamination. Despite the fact that intercropping snap beans with peppers can reduce the incidence of snap bean root rot, the precise mechanisms through which pepper root exudates confer this protective effect remain inadequately understood. This study demonstrates that pepper root exudates can effectively reduce the occurrence of snap bean root rot by reshaping the microbial community and inducing the secretion of plant defense metabolites. The significance of this research lies in elucidating the key mechanisms and application potential of pepper root exudates in enhancing snap bean resistance to root rot, thereby providing essential theoretical foundations and practical guidance for the development of novel biological agents and the advancement of green agriculture.

## INTRODUCTION

In agricultural ecosystems, continuous monoculture frequently leads to increased disease severity, a phenomenon closely associated with crop genetic uniformity and disruptions in the soil microbial community (1).

Root exudates play a crucial role in the rhizosphere ecosystem. On the one hand, root exudates supply abundant carbon and nitrogen sources, as well as energy substrates, thereby attracting and supporting the aggregation and proliferation of microorganisms in the rhizosphere (2–4). Besides, the composition and content of root exudates differ substantially among crop species, determining their specific attractiveness and selectivity for distinct microbes. For example, the root exudates of certain plants are enriched with specific sugars and amino acids, which facilitate the targeted recruitment of nitrogen-fixing bacteria, phosphate-solubilizing bacteria, or beneficial microbes that secrete plant hormones, thereby promoting their dominance within the rhizosphere microbial community (5–7). On the other hand, some secondary metabolites in root exudates possess inherent antimicrobial, bacteriostatic, or allelopathic properties, inhibiting the growth and proliferation of certain harmful microorganisms, thereby contributing to the regulation of rhizosphere microbial community balance and supporting the maintenance of healthy plant growth (8–10). Phenolic acid-like compounds secreted by the root system of wheat promote the inter-root colonization of *Streptomyces* spp. in pea and bolster resistance to pea wilt disease caused by *Fusarium oxysporum* spp. (11). Sulfur-containing compounds secreted by the root system of garlic promote the colonization of *Bacillus* spp. in the inter-root of peppers and inhibit the greening disease induced by *Ralstonia solanacearum* spp. (12). Furthermore, thioglucosides secreted by the root system of oilseed rape promote the proliferation of *Actinobacillus actinomycetemcomitans* and suppress *Rhizoctonia* spp. induced root rot in chickpea (13). These metabolites play crucial roles in enhancing plant immune responses and improving disease resistance. Crucially, such beneficial interactions mediated by root exudates are also evident in intercropping systems involving pepper (*Capsicum annuum*). For instance, pepper benefits from intercropping with rosemary through reduced pest pressures (14), while in a maize-pepper system, soil-borne disease suppression occurs via plant-plant-microbe mechanisms (15). These findings solidly establish pepper as both a recipient and a potential contributor in interspecific interactions. However, the specific role of pepper root exudates in enhancing the soil-borne disease resistance of snap beans remains unexplored.

*Phaseolus vulgaris L*. (snap bean) root rot limits yields wherever the crop is grown and constitutes a major constraint to dry edible and snap bean production worldwide (16). Reported root rot caused by *Fusarium solani* in the United States could cause yield losses of up to 84%. Reported yield losses from bean root rot complex are even more devastating in the developing world. Disease pressure is likely worse in developing countries because of higher abiotic stress. As a result of increased stress, bean root rot complex has been attributed to yield losses of up to 100% in Uganda and up to 70% in Rwanda (17). Cultural and chemical disease control methods are of limited value against root rots. Seed or soil treatments with selective fungicides, crop rotations, cover crops, seedbed preparations, and other measures have, in some cases, improved yield in the presence of *Aphanomyces euteiches* and *F. solani* root rot disease. However, few of these measures have proven consistently economical or effective against root rot (18). For the purpose of investigating the disease-suppressive effect of *Capsicum annuum L*. (pepper) root exudates on snap bean root rot and its microbiological mechanisms, root application of exogenous pepper root exudates was employed in this study to simulate their potential role within a natural intercropping system. The aim was to investigate whether the application of pepper root exudates alone could effectively mitigate root rot infection in snap beans by modulating the rhizosphere microbial community to recruit beneficial microorganisms, particularly those with plant disease-suppressive capabilities, and thereby effectively alleviate snap bean root rot infection. The following hypothesis was proposed: (i) exogenous application of pepper root exudates influences the structure, composition, and interspecies relationships of the snap bean rhizosphere microbial community; (ii) the exogenous application of pepper root exudates induces the biosynthesis of key metabolites; (iii) antagonistic microbes and key metabolites exhibit synergistic disease-suppressive effects. This study aims to elucidate the key mechanisms and application potential of pepper root exudates in enhancing the resistance of snap beans to root rot. We anticipate that this research will provide a novel biological strategy to reduce reliance on chemical fungicides, minimize environmental pollution, and contribute to the development of sustainable snap bean production systems based on rhizosphere ecological regulation.

## MATERIALS AND METHODS

### Soil and test material

The test soil was collected from the surface layer (0–15 cm) of a 3-year continuously cropped snap bean field at the experimental base of Heilongjiang Bayi Agricultural University, located in Daqing City, Heilongjiang Province, China. The soil type was black calcareous. Following collection, the soil was homogenized by passing it through a 2 mm sieve to remove stones and plant residues. Basic soil physicochemical properties were as follows: ammonium nitrogen 25.6 mg kg^−1^, nitrate nitrogen 40.4 mg kg^−1^, available phosphorus 11.6 mg kg^−1^, available potassium 114.7 mg kg^−1^, electrical conductivity (EC, 1:5 wt/vol) 0.92 mS cm^−1^, pH (1:2.5 wt/vol) 7.73.

The *F. solani* strain, isolated from infected snap bean plants, was cultured on potato dextrose agar (PDA) medium at 28°C as the pathogenic strain. The *F. solani* strain was prepared as a spore suspension (1 × 10^7^ conidia mL^−1^) for the pathogenic strain inoculation experiment. The experiment snap bean variety was “Heida Qingguan,” and the pepper variety was “Lameizi.” Seeds were surface-sterilized using 3% sodium hypochlorite for 3 min prior to sowing and subsequently rinsed three times with sterile deionized water.

### Experimental design

#### Pot experiment design

Snap beans were cultivated in 8  ×  7  ×  7  cm plastic pots, each filled with 200 g of non-sterilized soil. Four treatments were established: CK (no pepper root exudates, no *F. solani* inoculation), RE (pepper root exudates only), CKI (*F. solani* inoculation only), and REI (pepper root exudates + *F. solani* inoculation). For 4 treatments × 3 replications × 3 times (CK and RE treatments only taken on day 0), 15 pots were taken from each replication of each treatment, totaling 630 pots. Snap beans were grown in a greenhouse under controlled conditions of 25°C/22°C (day/night), 70% relative humidity, and a 12 h light/12 h dark photoperiod. Pots were randomly repositioned every 3 days to minimize experimental errors. Soil moisture was maintained at 70% of field capacity through irrigation with distilled water. The culture conditions were the same as in the determination of pepper root exudate dosage test. For the RE and REI treatments, 10 mL of pepper root exudate extract was applied to each pot every 48 h following seedling emergence, for a total of five applications. Rhizosphere soil and plant samples were collected 0 days after the fifth application (day of inoculation). On the same day, CKI and REI treatment snap bean roots were inoculated with 10 mL/pot of *F. solani* spore suspension (1 × 10⁷ conidia mL^−1^). Following *F. solani* inoculation, cultivation conditions were adjusted to day/night 28°C/25°C and 90% humidity. Rhizosphere soil and plant samples were collected at 5, 10, and 15 days post-inoculation. Snap bean roots were carefully removed from the pots, and the soil was removed by manually shaking the soil adhering to the roots. The tightly adhered soil was then removed from the root surface using a sterile brush as a rhizosphere soil sample. Rhizosphere soil was partitioned, with fresh samples utilized for microbial isolation and culture, while the remaining portion was stored at –80°C for microbial high-throughput sequencing analysis. Plant samples were collected for disease severity evaluation, biomass determination, and antioxidant enzyme activity assays.

### Antagonistic microbe isolation, culture, and efficacy validation experiment

One gram of rhizosphere soil from the 15 dpi REI treatment was suspended in 9 mL of 0.1% sterile sodium pyrophosphate solution and shaken at 200 rpm for 25 min. Following gradient dilution (10^−2^ to 10^−4^), aliquots were spread onto PDA plates (six replicates per dilution) and incubated at 25°C in the dark. Following isolation and purification of strains, antagonistic microbes were screened employing a dual-culture assay: *F. solani* was inoculated at the center of a PDA plate; after the colony expanded to 5 mm in diameter, isolated strains were inoculated 3.5 cm from the center. Furthermore, plates inoculated only with *F. solani* served as controls. After 7 days of incubation at 28°C, the inhibition rate (%) was calculated as [(Control radius − Treatment radius)/Control radius] × 100. After full-length sequencing of the 16S rRNA gene (primer 27F/1541R-new; PCR program: 94°C pre-denaturation for 5 min, 30 cycles [94°C 30 s, 55°C 45 s, 72°C 90 s], and final extension at 72°C for 10 min) (Jilin Comate Bioscience Co., Ltd.) and identified by NCBI BLAST comparison. The identified antagonistic strains were subsequently inoculated into the snap bean root system to verify their actual disease suppression.

Snap beans for the antagonistic validation test were planted in 8 × 7 × 7 cm plastic pots, each containing 200 g of sterilized soil. Four treatments were established: CK1 (no antagonist, no *F. solani*), Str (antagonist inoculation only), CKI1 (*F. solani* inoculation only), Str I (antagonist + *F. solani* inoculation). For 4 treatments × 3 replications, 15 pots were taken from each replication of each treatment, totaling 180 pots. Following snap bean emergence, each pot was inoculated with 10 mL of the antagonist suspension. Seven days later, CKI and REI treatment snap bean roots were inoculated with 10 mL/pot of *F. solani* spore suspension (1 × 10^7^ conidia mL^−1^) by root irrigation. Rhizosphere soil and plant samples were gathered 15 days after *F. solani* inoculation. Soil samples stored at −80°C were analyzed by DNA high-throughput sequencing for the determination of the relative abundance of *F. solani* and antagonists. Plant samples were utilized for disease evaluation and biomass determination. Culture conditions for snap beans were the same as in the pot experiment.

### Validation of disease-suppressive effect of flavone metabolite (Chrysin)

Based on untargeted metabolomics analysis, key disease-suppressive metabolites were identified and their disease-suppressing effects on root rot of snap bean were verified using potting experiments. Snap beans were cultivated in 8  ×  7  ×  7 cm plastic pots, each filled with 200  g of sterilized soil. Ten treatments were established: CK1 and CKI1 as defined in the antagonist validation assay, Chrysin only (CHR100, CHR500, CHR1000, CHR2000 μmol L^−1^), and Chrysin + *F. solani* (CHRI100, CHRI500, CHRI1000, CHRI2000 μmol L^−1^). Following snap bean emergence, 10 mL of Chrysin solution was administered to each pot at 48 h intervals, for a total of five applications. For 10 treatments × 3 replications, 15 pots were taken from each replication of each treatment, totaling 450 pots. Forty-eight hours after the fifth application, each pot was inoculated with 10 mL of *F. solani* spore suspension. Rhizosphere soil and plant samples were collected 15 days following *F. solani* inoculation. The rhizosphere soil from the most effective treatments (CHR1000, CHRI1000) and controls (CK1, CKI1) was stored at −80°C and analyzed by DNA high-throughput sequencing for the determination of the relative abundance of *F. solani* and antagonists. Plant samples were utilized for disease assessment and biomass measurement. Culture conditions for snap beans were the same as in the pot experiment.

### Collection and compositional analysis of pepper root exudates

Pepper seedlings with 6–7 true leaves were carefully uprooted and inoculated with *F. solani* for 15 days, followed by CK, RE, CKI, and REI treatments (pot trial) of snap bean seedlings, and their roots were thoroughly washed with sterile deionized water before being transferred into beakers covered with aluminum foil (containing 30 mL of sterile water per plant). The seedlings were then incubated at 28°C under light conditions for 6 h. The root exudate solution was filtered through a 0.45 µm membrane filter and used immediately. Transferred 100 uL filtrate to a 2 mL Eppendorf tube and resuspended with 400 µL extract solvent (acetonitrile–methanol, 1:1) by well vortex, then the sample was ultrasound for 10 min. The samples were incubated at −20°C for 1 h and centrifuged at 13,000 rpm and 4°C for 15 min. A volume of 350 µL supernatant was transferred to a 1.5 mL Eppendorf tube and then dried in a vacuum concentrator. The vacuum-dried sample was resolubilized with 1:1 acetonitrile–water, vortexed for 30 s, ultrasound for 10 min, then centrifuged at 13,000 rpm and 4°C for 15 min. Finally, take 50 µL of the supernatant and transfer it to an LC injection vial for metabolite component analysis. Ultra-high performance liquid chromatography (UHPLC) chromatographic separations were performed using an Acquity UHPLC system (Acquity LC, Waters) on a Waters UPLC column (ACQUITY UPLC BEH Amide 1.7 µm, 2.1 × 100 mm, Waters, Milford, MA). Mobile phase A was a solution consisting of 25 mM ammonium acetate and 25 mM ammonium hydroxide, and mobile phase B was 100% acetonitrile. The following gradient program was used: 95% B, 0.5 min; 95%–65% B, 0.5–7 min; 65%–40% B, 7–8 min; 40% B, 9 min; 40%–95% B, 9–9.1 min; 95% B, 12 min. The flow rate was 0.5 mL/min, the injection volume was 2 µL, and the samples were kept in the autosampler at 4°C. The UHPLC system was connected to a Q Exactive Hybrid Quadrupole-Orbitrap Mass Spectrometer (QE MS; Thermo Fisher Scientific, USA) system via an electrospray ionization source in positive ion mode. The following MS conditions were set: ion spray voltage (IonSpray Voltage Floating) of 5,500 V, ion source gas temperature of 650°C, ion source gas pressure of 60 psi, gas curtain gas pressure of 30 psi, and declustering voltage of 60 V. The QE MS data were acquired in the m/z range of 60−1,200 using a collision energy of 10 eV, and selecting the 12 most abundant mass spectral peaks in TOF MS for data-dependent acquisition scans. MS/MS data were recorded in the m/z 25−1,200 range using a collision energy of 30 eV and an acquisition rate of 0.03 s/spectrum.

### Determination of soil physicochemical indicators and disease incidence

#### Disease severity and disease index

The disease index was calculated based on the severity grade of root rot and the number of affected plants. The root rot severity grading criteria are as follows: Grade 0, no disease observed on snap bean roots; Grade 1, lesions covering less than 5% of the root surface area; Grade 2, lesions covering 6% to 10% of the root surface area; Grade 3, lesions covering 11% to 25% of the root surface area; Grade 4, lesions covering 26% to 50% of the root surface area; Grade 5, lesions covering 51% to 75% of the root surface area; Grade 6, lesions covering more than 75% of the root surface area or complete root rot.


$$\begin{array}{l} Disease\;index=[\Sigma \;(Number\;of\;diseased\;plants\;per\;grade\;\times \;Disease\;grade)\;/\;(Total\\ plants\;investigated\;\times \;Highest\;disease\;grade)]\;\times 100. \end{array}$$

Plants were weighed for fresh weight (FW) and dry weight (DW) using a balance, killed at 105°C and then baked at 65°C until constant weight.

Peroxidase (POD) activity was measured using the guaiacol method. Superoxide dismutase (SOD) activity was determined by the NBT method. Catalase (CAT) activity was assessed using the ultraviolet absorption method. Malondialdehyde (MDA) content was quantified by the TBA method. Furthermore, O₂⁻ production rate was measured using the hydroxylamine oxidation method. Proline (PRO) content was measured by utilizing the acid ninhydrin colorimetric method.

### High-throughput sequencing of 16S rRNA and ITS

Total genomic DNA was extracted from each sample using the E.Z.N.A. soil DNA kit (Omega Bio-tek, Norcross, GA, USA). Total DNA was extracted from soil samples according to the instructions. The quality and concentration of the DNA extract were assessed using a NanoDrop 2000 UV-vis spectrophotometer (Thermo Fisher Scientific, Wilmington, USA) and confirmed by running an agarose gel electrophoresis with 1% agarose gel. The V3–V4 region of the 16S rRNA gene was amplified for bacterial DNA assay using primers 338F (5′-ACTCCTACGGGGAGGCAGCAG-3′) and 806R (5′-GGACTACHVGGGTWTCTAAT-3′); the ITS1F (5′-CTTGGTCATTTAGAGGAAGTAA-3′) and ITS2R (5′-GCTGCGTTCTTCATCGATGC-3′) primers were used to amplify the ITS1 region for the assayed domains of fungal DNA. PCR products were recovered using a 2% agarose gel, purified using the DNA Gel Recovery and Purification Kit (PCR Clean-Up Kit, Passion, China), and quantified using Qubit 4.0 (Thermo Fisher Scientific, USA). The bacterial rRNA genes and fungal ITS genes were subjected to high-throughput sequencing using the Illumina MiSeq PE300 platform (Illumina, San Diego, USA) at Majorbio Bio-Pharm Technology Co., Ltd. (Shanghai, China). Standard protocols were followed during the sequencing process to ensure the accuracy and reliability of the data.

### Bioinformatics and analysis

The raw sequencing reads of the 16S rRNA and ITS genes were processed using fastp version 0.20.0 for demultiplexing and quality filtering. Subsequently, the filtered reads were merged using FLASH version 1.2.7. Operational taxonomic unit (OTU) clustering was conducted using UPARSE version 7.1 at a 97% similarity threshold on the non-redundant sequences, excluding single sequences. Chimeric sequences were effectively removed during the clustering process. The OTU table was manually filtered, i.e., chloroplast sequences in all samples were removed. The taxonomy of each OTU representative sequence was analyzed by RDP Classifier version 2.2 against the 16S rRNA gene database (Silva v138, http://www.arb-silva.de), ITS gene database (Unite v8.0, http://unite.ut.ee/index.php) using a confidence threshold of 0.7.

Significance of differences between multiple groups of data was analyzed using analysis of variance or the Kruskal-Wallis test. Bar graphs were plotted using GraphPad Prism 9. Microbial alpha-diversity indices were computed using the “phyloseq” package in R (v.3.6.2). The similarity of microbial community structure between samples was examined using principal coordinate analysis (PCoA) based on the Bray-Curtis distance algorithm and combined with the permutational multivariate analysis of variance non-parametric test. The Mean Decrease Gini index derived from the Random Forest algorithm was employed as the metric for evaluating feature importance. Partial least squares discriminant analysis (PLS-DA, VIP > 1) was utilized to screen for differential metabolites.

## RESULTS

### Exogenous pepper root exudates attenuate root rot occurrence and activate plant immune responses

**Fig 1:**
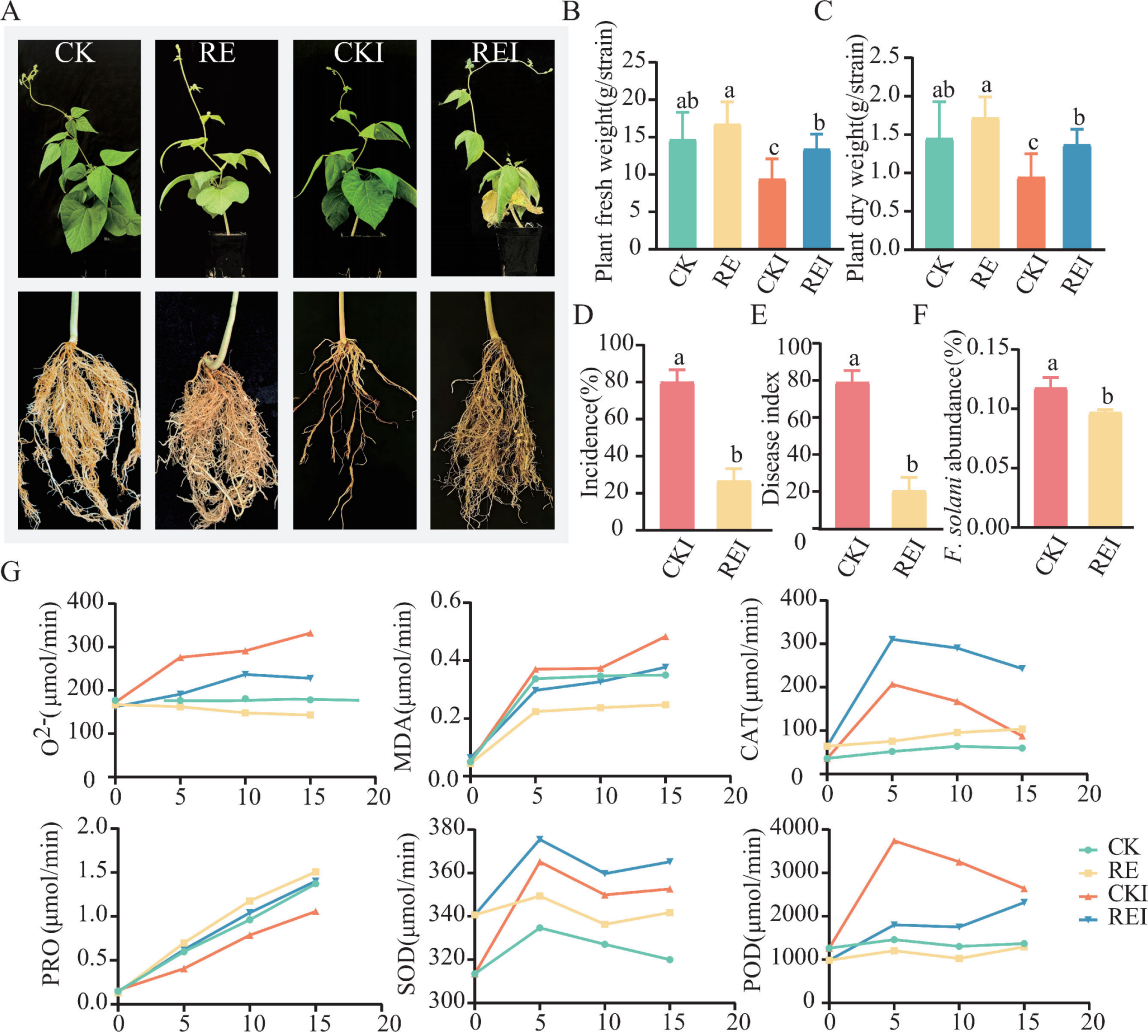
Disease control effect of pepper root exudates on snap bean root rot and plant immune responses. (**A**) Growth status of CK, RE, CKI, and REI snap bean plants. (**B**) Fresh biomass of CK, RE, CKI, and REI snap bean plants. (**C**) Dry biomass of CK, RE, CKI, and REI snap bean plants. (**D**) Disease incidence of root rot in CKI and REI snap bean plants. (**E**) Disease index of root rot in CKI and REI snap bean plants. (**F**) Relative abundance of *F. solani* in the rhizosphere soil of CKI and REI snap bean plants. (**G**) Changes in antioxidant indicators in the rhizosphere soil of CK, RE, CKI, and REI snap bean plants. CK: no pepper root exudates, no *F. solani*; RE: pepper root exudates only; CKI: *F. solani* inoculation only; REI: pepper root exudates + *F. solani* inoculation.

Pot experiments demonstrated that exogenous application of pepper root exudates significantly inhibited *F. solani*-induced snap bean root rot (Fig. 1A) (19). Fifteen days after *F. solani* inoculation, CKI-treated plants demonstrated obvious wilting, while REI-treated plants grew normally. In comparison to CK, plant dry weight and fresh weight values were 18% and 14% higher in the RE treatment, but there were no significant differences. Compared to CKI, plant dry weight and fresh weight values were 53% and 44% higher in the REI treatment, respectively (Fig. 1B and C). Indicating that pepper root exudates partially alleviated the growth inhibition induced by pathogen stress. The root rot disease index and incidence rate treated with REI were 47% and 53.3% lower than CKI (Fig. 1D and E). 16S rRNA results further confirmed that the relative abundance of *F. solani* in REI-treated snap bean rhizosphere soil was substantially lower than in CKI (Fig. 1F). The small proportion of *F. solani* in the community may indicate that *F. solani* may have been suppressed, but it is also possible that the growth of other organisms reduced the relative abundance of *F. solani*, even though *F. solani* was still growing.

Pathogen infection caused oxidative stress in snap bean plants, and the contents of superoxide anion (O^2-^) and MDA in CKI leaves were 15.2-fold and 9.5-fold higher than that in REI (Fig. 1G). The activities of CAT, SOD, and POD in REI were 0.3-fold, 0.38-fold, and 1.77-fold higher than that of CKI (Fig. 1G). PRO content was abnormally low in CKI, being 1.14-fold, 1.62-fold, and 1.24-fold lower than in CK, RE, and REI, respectively, suggesting that *F. solani* stress may disrupt proline metabolism.

### Pepper root exudates drive microbial community restructuring

**Fig 2:**
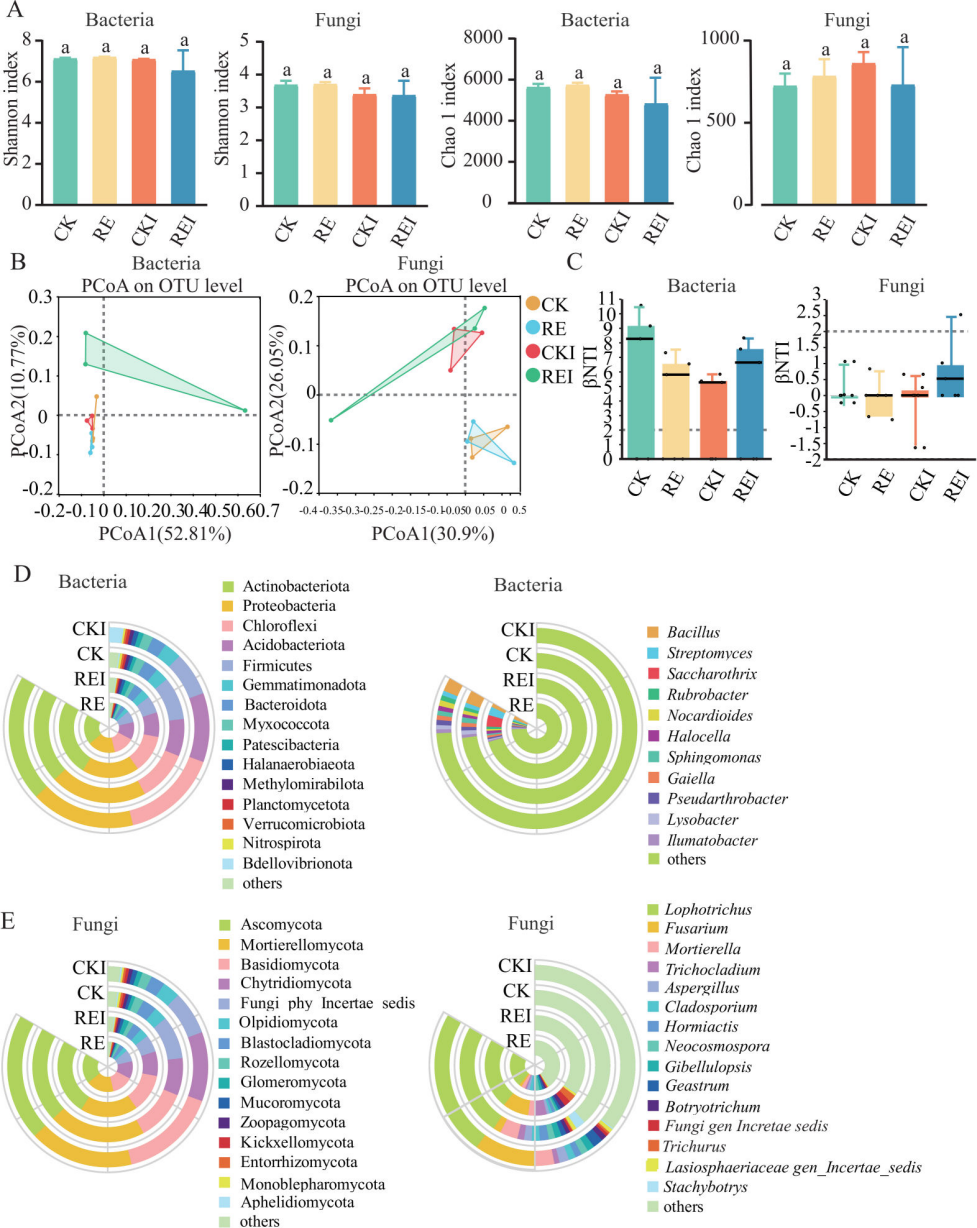
Microbial community diversity and structural changes across treatments. (**A**) Alpha diversity of bacterial and fungal communities. (**B**) PCoA of bacterial and fungal communities. (**C**) Community assembly model (βNTI) for bacterial and fungal communities. (**D**) Dominant bacterial phyla and genera. (**E**) Dominant fungal phyla and genera. CK: no pepper root exudates, no *F. solani*; RE: pepper root exudates only; CKI: *F. solani* inoculation only; REI: pepper root exudates + *F. solani* inoculation.

The Shannon and Chao1 indices of bacterial and fungal communities in rhizosphere soil showed no significant differences among treatments (Fig. 2A). However, PCoA revealed a distinct separation of REI samples from those of the other treatments, indicating a marked shift in microbial community structure, indicating that *F. solani* inoculation significantly altered bacterial and fungal community structure (Fig. 2B). Bacterial communities in all treatments were strongly dominated by heterogeneous selection (|βNTI| > 2), demonstrating high dependence on environmental filtering for community assembly. Bacterial βNTI in the REI group was substantially higher than in CKI (7.508 > 5.486), suggesting pepper root exudates strengthened deterministic selection under *F. solani* stress. The influence of pepper root exudates (RE) on the fungal community was weaker than on the bacterial community, with only a subset of samples exhibiting deterministic selection; overall, stochastic processes remained predominant (Fig. 2C).

In the bacterial community, Actinobacteriota, Proteobacteria, Chloroflexi, Acidobacteriota, *Bacillus*, and *Streptomyces* were the dominant phyla and genera in the four treatments. Among them, Actinobacteria (24.5%) and Proteobacteria (25%) were dominant phyla in CK (Fig. 2D). In comparison to CKI, REI increased the relative abundance of Actinobacteria (29.5% vs 24.4%), Bdellovibrionota (3.7% vs 3.4%), and specifically enriched *Streptomyces* (3.3% vs 0.9%), *Saccharothrix* (4.5% vs 0.004%), and *Halocella* (1.2% vs 0.9%) (Fig. 2D). Notably, compared to CK, CKI increased the relative abundance of p__Chloroflexi (18.7% vs 13.1%) and Acidobacteriota (13.4% vs 9.7%), which illustrates a key role for pepper root exudates in regulating rhizosphere microecological balance, potentially enhancing plant disease resistance by optimizing the microbial community structure.

Fungal community analysis indicated that Ascomycota, Mortierellomycota, Basidiomycota, Chytridiomycota, and *Lophotrichus* were the dominant phyla and genera in the four treatments. Among them, Ascomycota (84.3%) was the dominant phylum in the CK treatment. Compared to CKI, REI reduced the relative abundance of *Lophotrichus* (27.3% vs 28.2%) and increased the relative abundance of *Trichocladium* (4.8% vs 1.0%) and *Botryotrichum* (1.6% vs 1.1%) (Fig. 2E). Moreover, CKI increased *Fusarium* abundance (11.8%), while the REI treatment suppressed it to 10.1% (Fig. 2E). This result is closely related to the observed decrease in the root rot disease index (Fig. 1E), indicating that pepper root exudates are one of the reasons for reducing the occurrence of root rot disease in snap bean by inhibiting the proliferation of *F. solani*.

The application of pepper root exudates significantly optimized rhizosphere microecological function. In the bacterial community, the m-value in REI was notably lower than in CKI (0.8383 vs 1.0302), indicating that pepper root exudates enhanced deterministic community assembly driven by environmental selection. This was likely achieved by reducing pathogen abundance, suppressing the proliferation of harmful bacteria, and promoting the stability of disease resistance-associated microbiota. In contrast, m-values in the fungal community were consistently low (<0.12) across treatments, suggesting that fungal community assembly was primarily governed by stochastic processes. Although REI did not significantly alter the fungal assembly pattern (m = 0.0906 vs CKI 0.1159), it preserved fungal functional stability, potentially through conserved substrate interactions that support synergistic disease resistance (Fig. S1).

### Bacterial–fungal relationships

**Fig 3:**
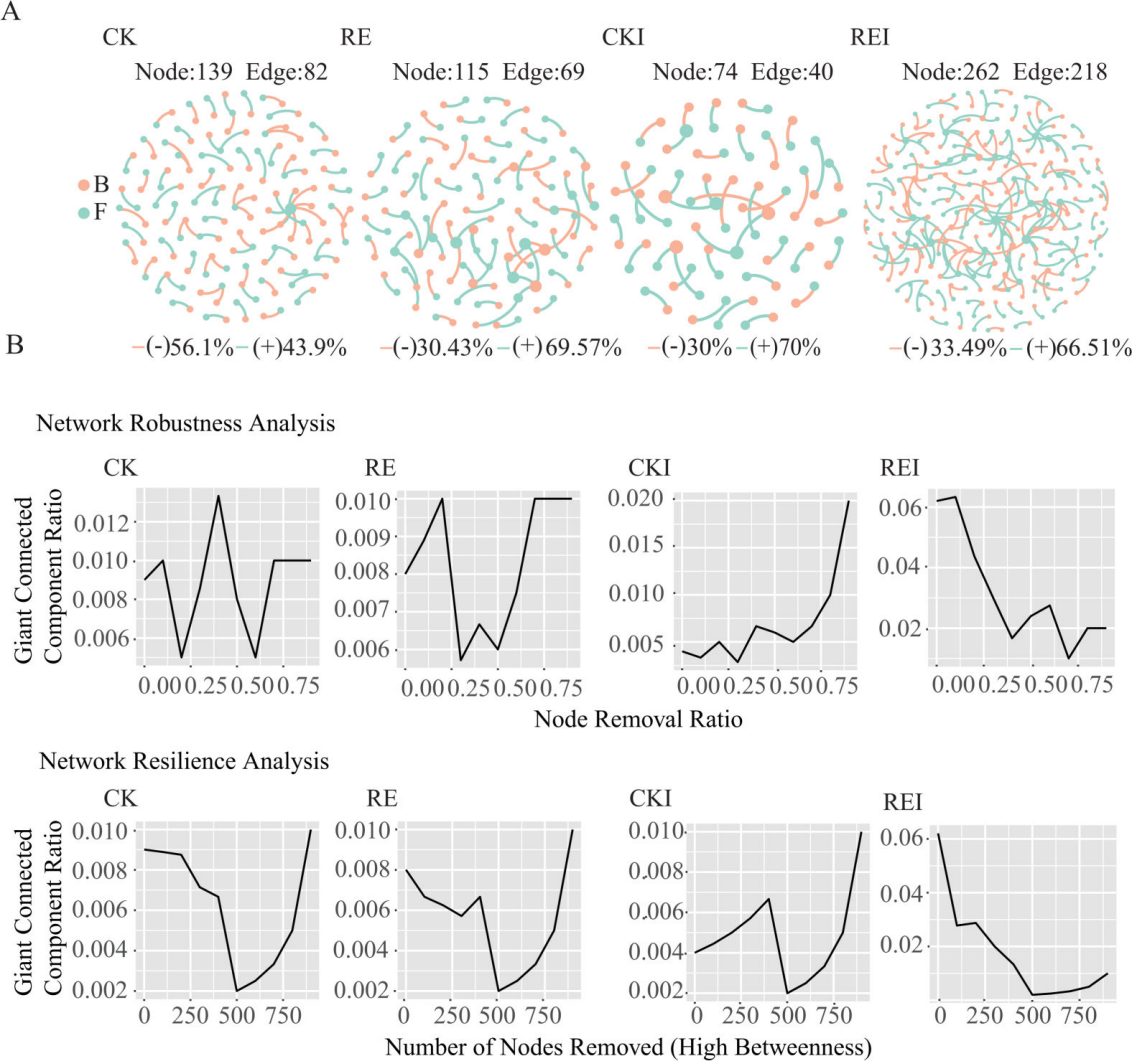
Bacterial–fungal cross-domain co-occurrence network analysis for CK, RE, CKI, and REI. (**A**) Bacterial–fungal cross-domain networks for CK, RE, CKI, and REI. (**B**) Robustness and resilience of CK, RE, CKI, and REI networks. CK: no pepper root exudates, no *F. solani*; RE: pepper root exudates only; CKI: *F. solani* inoculation only; REI: pepper root exudates + *F. solani* inoculation; B: bacteria; F: fungi.

Analysis of bacterial–fungal relationships and network properties across the four treatments (CK, RE, CKI, REI) using cross-domain networks (Fig. 3A) revealed that the REI treatment exhibited higher robustness and resilience, indicating improved network stability. Additionally, a higher closeness centrality value (0.16) (Table S1) suggested greater efficiency in information transfer between nodes and a more compact network structure. In contrast, CKI displayed the highest number of components (960), reflecting greater network fragmentation and reduced stability. Regarding network complexity, REI showed significantly higher values for degree (0.436), number of edges (219), and number of nodes (262) compared to the other treatments, indicating a more densely interconnected microbial network. Conversely, CKI had lower values for degree (0.08), edges (40), and nodes (70), indicating lower network complexity. Application of pepper root exudates (RE and REI) substantially enhanced network complexity and stability, particularly under *F. solani* inoculation (REI). This suggests pepper root exudates may enhance network resilience and functional diversity by promoting microbial interactions.

### Screening of important microbes and analysis of their relationship with *Fusarium*

**Fig 4:**
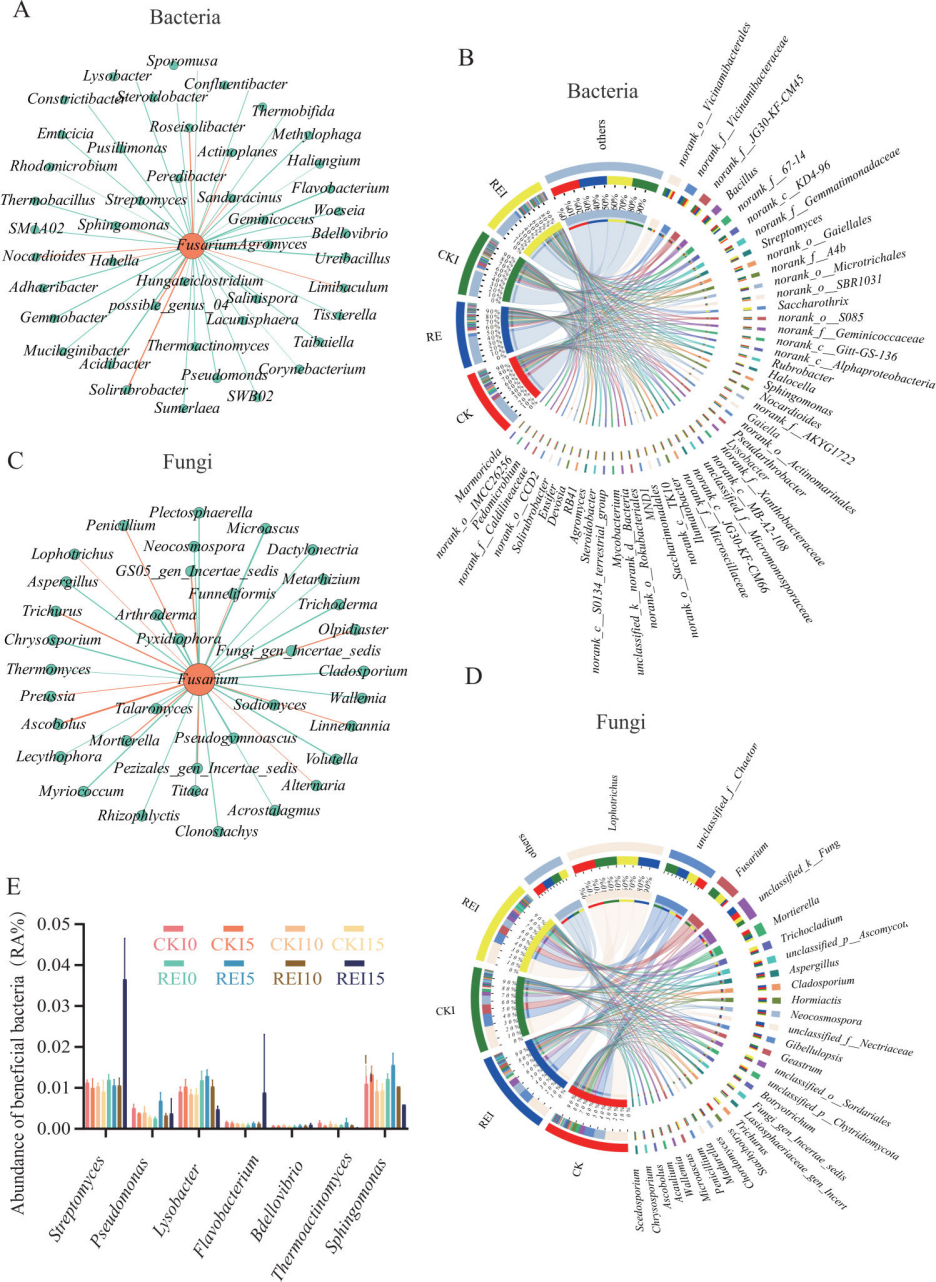
Bacterial and fungal genera of their relationship with *Fusarium*. (**A**) Correlation between important bacterial genera and *Fusarium*. (**B**) Abundance of dominant bacterial genera in CK, RE, CKI, and REI at 15 days post-*Fusarium* inoculation. (**C**) Correlation between important fungal genera and *Fusarium*. (**D**) Abundance of dominant fungal genera in CK, RE, CKI, and REI at 15 days post-*Fusarium* inoculation. (**E**) Changes in relative abundance of beneficial bacterial genera in CK, RE, CKI, and REI treatments at 0, 5, 10, 15 days. CK: no pepper root exudates, no *F. solani*; RE: pepper root exudates only; CKI: *F. solani* inoculation only; REI: pepper root exudates + *F. solani* inoculation.

The Mean Decrease Gini index derived from the Random Forest algorithm was employed as the evaluation metric for OTU feature importance. The top 50 most important bacterial OTUs and fungal OTUs (Fig. S2) were ranked in descending order based on their Mean Decrease Gini values. The relationships between the bacterial and fungal genera represented by these essential OTUs and *Fusarium* were analyzed. In the bacterial network (Fig. 4C), nine genera indicated significant positive correlations with *Fusarium: Actinoplanes*, *Limibaculum*, *Sandaracinus*, *Agromyces*, *possible_genus_04*, *Roseisolibacter*, *Solirubrobacter*, *Nocardioides*, and *Hahella*. Forty bacterial genera indicated significant negative correlations with *Fusarium*, including *Pseudomonas*, *Lysobacter*, *Flavobacterium*, *Bdellovibrio*, *Thermoactinomyces*, *Streptomyces*, and *Sphingomonas*, which are associated with resistance to plant fungal diseases (20) (Fig. 4A). Species composition analysis (Fig. 4B) revealed that, relative to CKI, REI treatment significantly increased the abundance of *Streptomyces* (50% vs 14%), *Saccharothrix* (100% vs 0%), *Halocella* (33%vs 25%), *Mycobacterium* (34% vs 23%), *Steroidobacter* (41% vs 18%), *Devosia* (50% vs 18%), and *Ensifer* (30% vs 21%), while their abundance decreased significantly in CK and CKI treatments.

In the fungal network (Fig. 4C), 38 genera indicated significant positive correlations with *Fusarium*, including *Lophotrichus*, *Trichurus*, *Linnemannia*, *Mortierella*, *Alternaria*, *Funneliformis*, etc., associated with fungal disease induction (21). Twelve fungal genera showed significant negative correlations with *Fusarium*, including *Metarhizium*, *Clonostachys*, and *Trichoderma*, associated with resistance to plant fungal diseases (22). Species composition analysis (Fig. 4D) demonstrated that, compared to CKI, the REI treatment significantly increased the abundance of *Trichocladium* (53% vs 11%), *Botryotrichum* (24% vs 21%), *Trichurus* (75% vs 11%), *Microascus* (59% vs 7%), and *Arcuadendron* (47% vs 12%), while their abundance decreased substantially in CK and CKI treatments. The relative abundance of bacteria associated with resistance to plant fungal diseases, including *Pseudomonas*, *Lysobacter, Flavobacterium*, *Bdellovibrio*, *Thermoactinomyces*, *Streptomyces,* and *Sphingomonas,* at 0, 5, 10, and 15 days was displayed in bar charts (Fig. 4E). It was observed that the relative abundance of *Streptomyces* in REI was higher than in CKI at 0, 5, 10, and 15 days.

### Screening of antagonistic microbes and validation of their disease resistance

**Fig 5:**
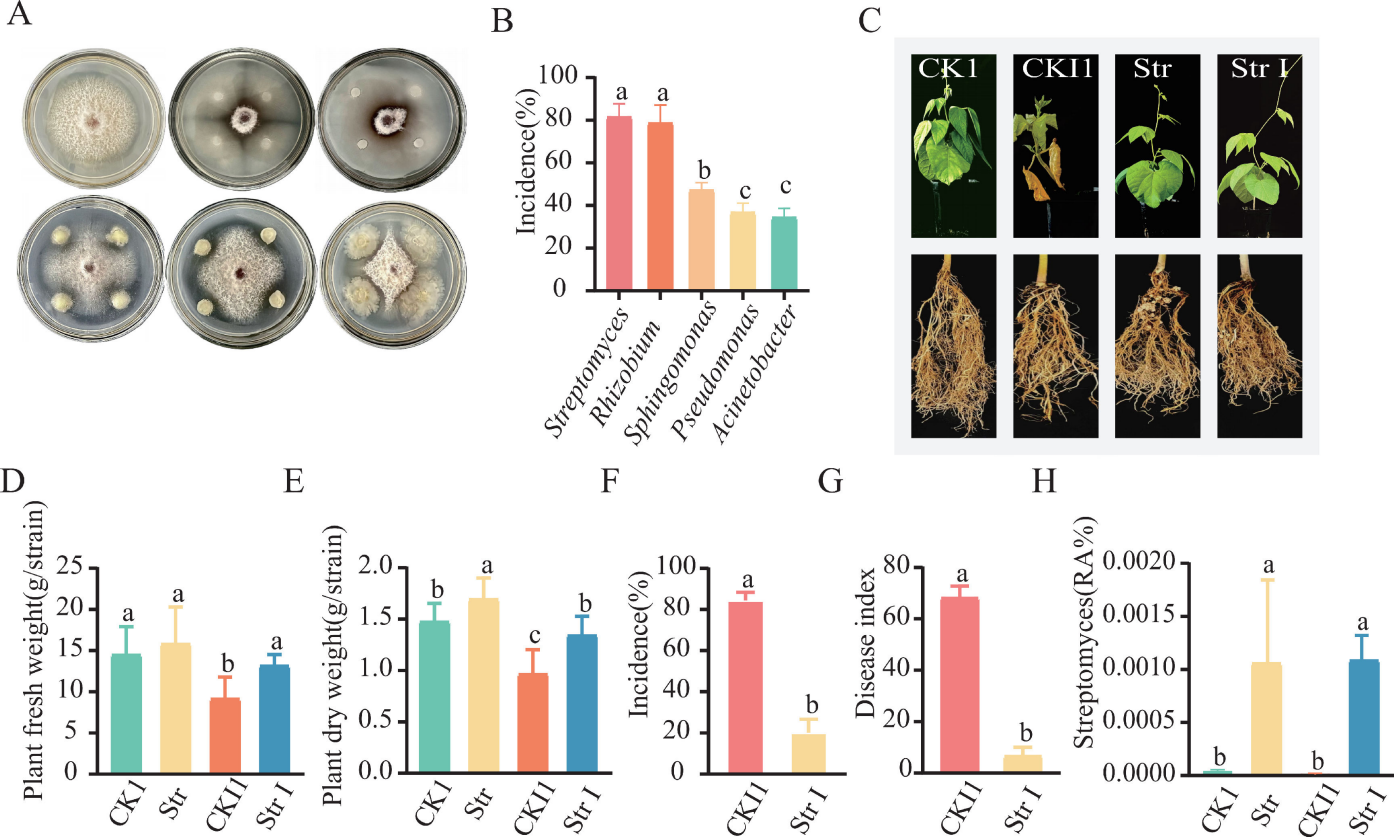
Antifungal function of *Streptomyces* and re-inoculation validation assay. (**A**) Dual culture of isolated antagonists against *F. solani*. (**B**) Inhibition rate of antagonists against *F. solani* hyphal growth. (**C**) Growth status of snap bean plants (CKI1, Str I) after *F. solani* inoculation. (**D**) Fresh biomass of CK1, CKI1, Str, and Str I snap bean plants. (**E**) Dry biomass of CK1, CKI1, Str, and Str I snap bean plants. (**F**) Disease incidence of root rot in CKI1 and Str I snap bean plants. (**G**) Disease index of root rot in CKI1 and Str I snap bean plants. (**H**) Relative abundance of *Streptomyces* in rhizosphere soil of CK1, CKI1, Str, and Str I snap bean plants. CK1: no antagonist, no *F. solani*; Str: antagonist inoculation only; CKI1: *F. solani* inoculation only; Str I: antagonist + *F. solani* inoculation.

A total of 73 strains were isolated and cultured from REI rhizosphere soil samples using PDA medium. Dual-culture assays against *F. solani* with these 73 strains demonstrate that 19 strains exhibited varying degrees of antagonism. These 19 antagonistic strains were submitted to Comate Bioscience Co., Ltd. (City, China) for identification, resulting in the identification of five antagonistic strains: *Streptomyces*, *Rhizobium*, *Sphingomonas*, *Pseudomonas*, and *Acinetobacter* (Fig. 5A) (Table S1). Their inhibition rates against *F. solani* hyphal growth ranged from 34.43% to 81.43% (Fig. 5B). To further confirm the disease-suppressive efficacy of these five antagonists, they were inoculated onto the roots of snap bean plants cultivated in sterile soil. Plants inoculated with *Streptomyces* showed the best growth and mildest root rot symptoms, largely alleviating disease symptoms (Fig. 5C). Furthermore, the dry and fresh weight of snap bean plants (Str I) inoculated with *Streptomyces* were substantially higher than in CKI1 (Fig. 5D and E). Compared to CKI1, Str I treatment significantly decreased the snap bean root rot disease index by 61.7% and disease incidence by 64.47% (Fig. 5F and G). 16S RNA results showed significant enrichment of *Streptomyces* in the rhizosphere soil of Str and Str I treatments. Combined with the results of antagonistic microbe isolation culture and efficacy validation experiments, *Streptomyces* was identified as the most effective antagonist against *F. solani*-induced snap bean root rot (Fig. 5H).

### Validation of disease-suppressive effect of key metabolite (Chrysin)

**Fig 6:**
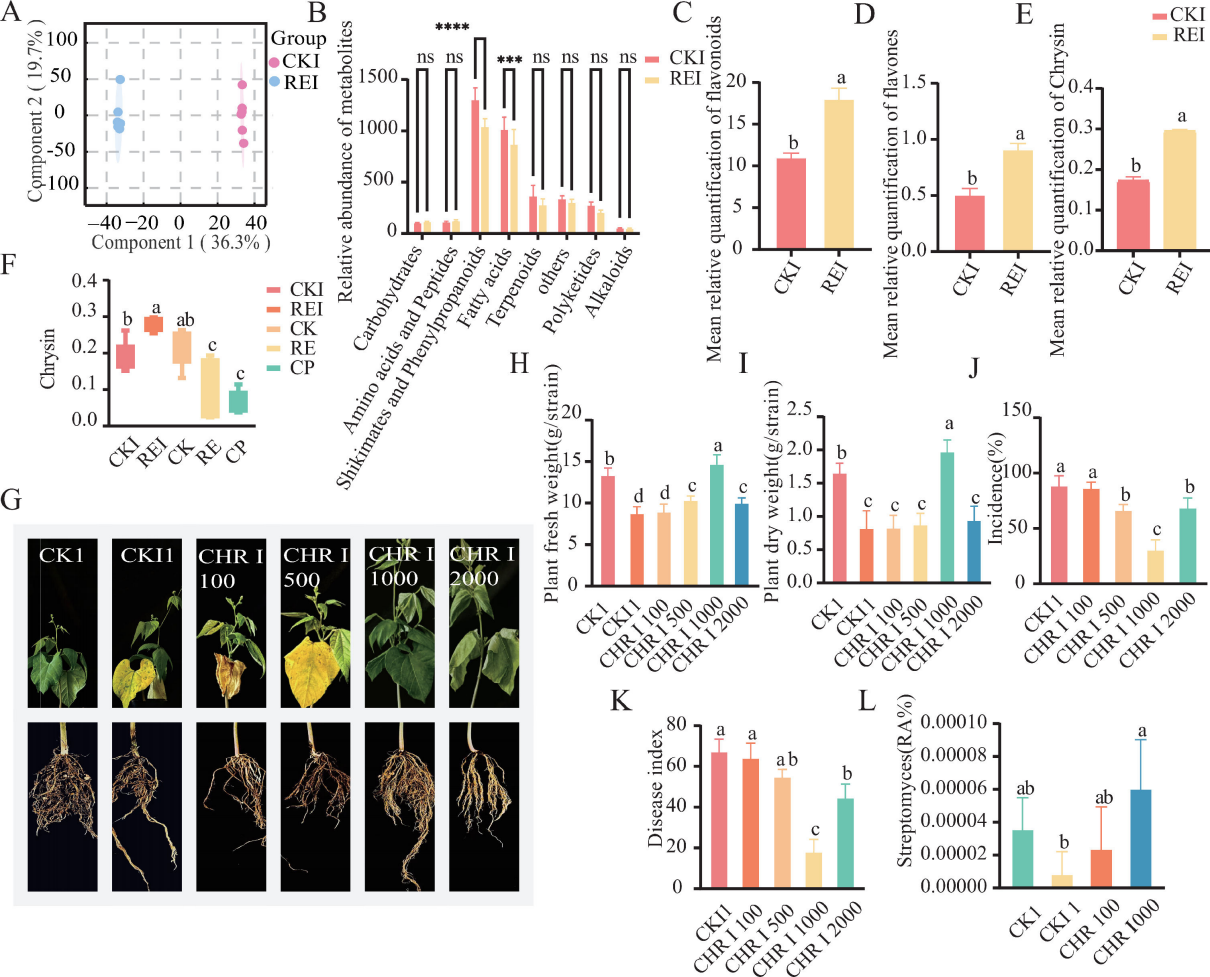
Differential analysis of flavonoid metabolites mediated by pepper root exudates. (**A**) OPLS-DA of snap bean (CKI, REI) metabolites. (**B**) Relative abundance of secondary classification metabolites in CKI and REI treatments. (**C**) Relative abundance of tertiary classification flavonoid metabolites in CKI and REI treatments. (**D**) Relative abundance of quaternary classification flavone metabolites in CKI and REI treatments. (**E**) Relative abundance of the flavone metabolite Chrysin in CKI and REI treatments. (**F**) CK, RE, CKI, REI, and CP treated with relative content of Chrysin. (**G**) Growth of snap bean inoculated with *F. solani* (CHRI 100, CHRI 500, CHRI 1000, CHRI 2000) following exogenous Chrysin treatment. (**H**) Fresh biomass of snap bean plants (CK1, CKI1, CHRI 100, CHRI 500, CHRI 1000, CHRI 2000). (**I**) Dry biomass of snap bean plants (CK1, CKI1, CHRI 100, CHRI 500, CHRI 1000, CHRI 2000). (**J**) Disease incidence of root rot in snap bean plants (CKI1, CHRI 100, CHRI 500, CHRI 1000, CHRI 2000). (**K**) Disease index of root rot in snap bean plants (CKI1, CHRI 100, CHRI 500, CHRI 1000, CHRI 2000). (**L**) Relative abundance of *Streptomyces* in rhizosphere soil of snap bean plants (CK1, CKI1, CHRI 100, CHRI 500, CHRI 1000, CHRI 2000). CK1: no Chrysin, no *F. solani*; CHR: Chrysin only at indicated concentration; CKI1: *F. solani* inoculation only; CHRI: Chrysin at indicated concentration + *F. solani* inoculation.

Orthogonal partial least squares discriminant analysis (OPLS-DA) revealed significant differences (*P* < 0.05) in the composition of root metabolites between CKI and REI-treated snap bean plants (Fig. 6A). Within the HMDB secondary classification, significant variations were observed in shikimates, phenylpropanoids, and fatty acids between CKI and REI (Fig. 6B). At the tertiary classification level, flavonoids exhibited notable changes, with their average relative content increasing from 11.03% in CKI to 18.03% in REI, representing a 63.5% rise (Fig. 6C). At the quaternary classification level, flavones also showed significant differences, with REI content being 78.4% higher than that of CKI (Fig. 6D), and Chrysin content was significantly elevated in REI (Fig. 6E). According to the untargeted metabolomics analysis, the RE and CP treatments had lower Chrysin itself and the CK treatment had higher Chrysin itself, but the REI treatment had significantly higher Chrysin content than the CKI treatment after accessing *F. solani*. It was shown that pepper root exudates promoted flavones accumulation in snap beans after accessing *F. solani* (Fig. 6F). Eight compounds were identified within the flavones category: Swertiajaponin, 2-(2,6-dimethoxyphenyl)-5,6-dimethoxy-chromen-4-one, Diosmin, Chrysin, Camellianin A, Diosmetin_7-O-beta-D-glucuronopyranoside, (9R,10S)-rel-(-)-9,10-bis(acetyloxy)-9,10-dihydro-5-methoxy-8,8-dimethyl-2-phenyl-4H,8H-benzo[1,2-b:3,4-b']dipyran-4-one, Nobiletin, Luteolin 7-O-[beta-D-glucuronosyl-(1→2)-beta-D-glucuronide]. Diosmin (molecular weight >600 Da, specifically Diosmetin\_7-O-glucuronide at 640.5 Da) is too large to effectively penetrate fungal cell walls. Glycosylated flavonoids, such as Camellianin A, exhibit significantly increased molecular polarity (logP < −1.2) due to sugar side chains, which reduces their capacity for transmembrane transport; moreover, there have been no recent reports of their antifungal activity. The complex dimeric structure of (9R,10S)-rel derivatives (molecular weight 534.5 Da) presents synthetic challenges, and fungal target prediction software (PhytoPathPred) indicates no binding affinity to key fungal proteins, such as CYP51. QSAR models predicted low antifungal activity indices (AFI < 0.35) for other methoxy-containing derivatives. Moreover, Chrysin has an EC50 <50 µg/mL against plant pathogenic fungi, its molecular weight (254.24 Da) complies with Lipinski’s rule (23, 24), indicating favorable bioavailability potential. To assess the biocontrol efficacy of Chrysin against snap bean root rot, a re-inoculation validation assay was performed through pot experiments. Results demonstrated that compared to CKI1, CHRI 1000-treated plants grew well without obvious wilting, while CHRI 100, CHRI 500, and CHRI 2000 plants indicated significant wilting (Fig. 6G). Fresh and dry biomass of CHRI 1000-treated plants were 1.67-fold and 2.38-fold greater, respectively, than those of CKI1 (Fig. 6H and I). Compared to CKI1, CHRI 1000 treatment significantly reduced the root rot disease index by 49.4% and disease incidence by 57.8% (Fig. 6J and K). 16S RNA results showed significant enrichment of *Streptomyces* in CHRI 1000 rhizosphere soil, indicating that exogenous application of Chrysin recruits Streptomyces snap bean rhizosphere enrichment in the event of pathogen infection, which is friendly to the effective mitigation of root rot (Fig. 6L).

### Microbial-metabolite synergistic disease resistance mechanism

**Fig 7:**
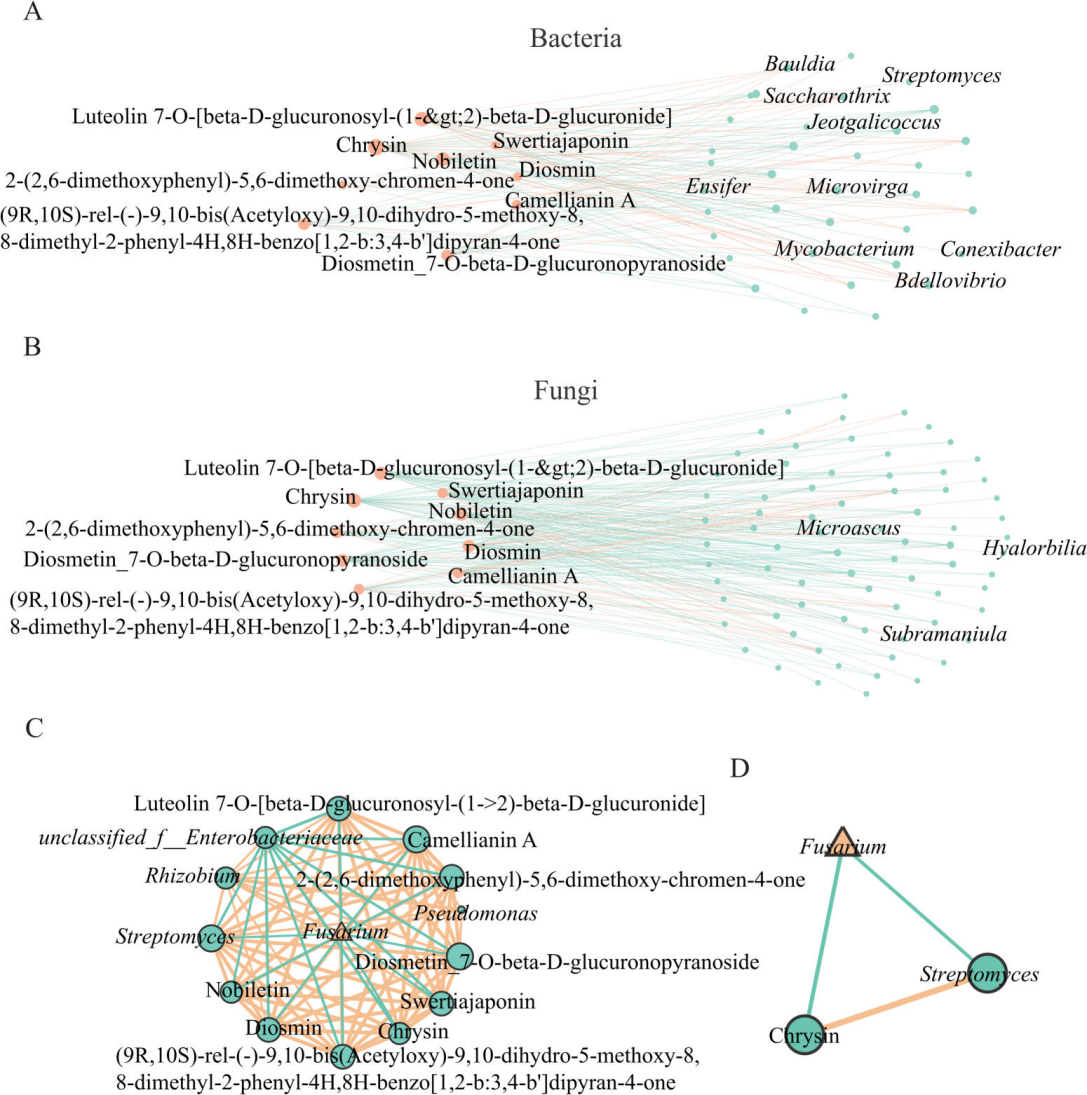
Interaction network among flavone metabolites, *Fusarium*, and antagonistic genera. (**A**) Interaction network between flavone metabolites and bacterial genera. (**B**) Interaction network between flavone metabolites and fungal genera. (**C**) Interaction network among flavone metabolites, *Fusarium*, and antagonistic genera. (**D**) Interaction network among Chrysin, *Fusarium*, and Streptomyces. (**A, B**) Orange points: flavone metabolites; green points: bacterial/fungal genera. (**C, D**) Orange triangle: *Fusarium*; green points: flavone metabolites/antagonistic genera. Orange lines: positive correlation; green lines: negative correlation.

Spearman correlation analysis between quaternary-classified flavone metabolites and the top 200 bacterial and fungal genera revealed positive correlations with the majority of bacterial genera and negative correlations with most fungal genera (Fig. 7A and B). Figure 7 indicates that Chrysin positively regulates *Streptomyces*, *Ensifer*, *Jeotgalicoccus*, *Mycobacterium*, *Conexibacter*, *Bauldai*, *Microvirga*, *Bdellovibrio*, and *Saccharothrix*, while *Saccharothrix* and *Streptomyces* were negatively correlated with *Fusarium*. Figure 7B demonstrates that Chrysin positively regulates *Microascus*, *Hyalorbilia*, and *Subramaniula*, among which *Microascus* was negatively correlated with *Fusarium*. Network analysis between flavone metabolites, *Fusarium*, and the screened antagonistic genera (Fig. 7C and D) revealed that *Fusarium* was negatively correlated with *Streptomyces* and Chrysin, while *Streptomyces* was positively correlated with Chrysin.

## DISCUSSION

This study investigated the synergistic mechanisms between microbes and metabolites involved in the suppression of *Fusarium solani-*induced snap bean root rot through the exogenous application of pepper root exudates. Pot experiments demonstrated that exogenous pepper root exudates effectively mitigated snap bean root rot occurrence. Furthermore, under *F. solani* infection, exogenous pepper root exudates selectively enriched beneficial microbes (e.g., *Streptomyces*, *Saccharothrix*, *Microascus*) in snap bean rhizosphere soil while inducing the accumulation of endogenous plant flavonoid metabolites, forming a dual defense barrier. Plate confrontation experiment, antagonistic bacteria, and key metabolite validation test further confirmed the disease-suppressive effects of antagonistic microbes and key metabolites, such as Chrysin.

### Exogenous pepper root exudates attenuate root rot occurrence by activating plant immune responses

This study determined that exogenous pepper root exudates substantially reduced snap bean root rot severity and *F. solani* relative abundance while increasing plant dry/fresh weight and antioxidant enzyme (CAT, SOD, POD) activities (Fig. 1). The enhanced antioxidant enzyme activity observed in REI may be associated with the reactive oxygen species (ROS) signaling pathway. SOD and CAT function cooperatively to scavenge ROS and maintain cellular redox homeostasis (25). Previous studies indicate root exudates can enhance defense by activating systemic acquired resistance (26). Additionally, phenolic acids in tomato root exudates can induce antioxidant enzyme activity, mitigating oxidative stress damage (27). Notably, *F. solani* infection resulted in abnormal decreases in PRO content, while REI treatment substantially restored its homeostasis (Fig. 1G). Disruption of proline metabolism may represent an adaptive strategy by which plants reallocate resources toward defense pathways under stress conditions. This study demonstrates that pepper root exudates significantly suppress *F. solani* proliferation, thereby mitigating snap bean root rot.

### Structural remodeling of the microbial community and cross-domain synergistic disease resistance mechanism

At the level of community structure, REI treatment significantly enriched Actinobacteria, especially *Streptomyces* and *Saccharothrix*, while reducing Proteobacteria, especially *Bacillus*. These taxa are antibiotic and secondary metabolite producers with a strong ability to antagonize pathogenic bacteria (28). This phenomenon is consistent with the mechanism of selective enrichment of actinomycetes by maize root exudates through phenolic acids (29). Flavonoids present in pepper root exudates may enhance the antagonistic activity of Actinobacteria by activating antibiotic biosynthesis gene clusters, such as PKS and NRPS (30), while promoting their metabolism of specific carbon sources. Besides, the reduction of γ-Proteobacteria may stem from direct inhibition of harmful bacteria by root exudates while promoting competition by beneficial bacteria (31). The reduction of *Bacillus* genera may reflect resource competition triggered by the enrichment of strong antagonists (32), confirming the functional redundancy of the microbial community. This phenomenon suggests that the disease resistance function of microbial communities is not dominated by a single genus, but is the result of the synergistic action of multiple taxa.

In the fungal community, REI treatment reduced the relative abundance of *F. solani*, suggesting that root exudates may exert an inhibitory effect by directly inhibiting *F. solani* spore germination (33). REI treatment promoted an increase in the relative abundance of *Trichoderma,* which may induce plant systemic resistance through the secretion of active substances, such as chitinase (34). REI treatment reduced the relative abundance of *Mortierellomycota,* possibly because root exudates inhibited saprophytic bacteria and enriched disease-resistant functional flora through phenolics (35).

Cross-domain network analysis showed that the REI treatment formed the most complex, stable, tightly connected, and least fragmented microbial co-occurrence network relative to the CKI treatment. It indicated that pathogen infestation disrupted the original microbial interactions and formed a fragile and easily disturbed community state, which was conducive to the proliferation of pathogenic bacteria and disease development. The root exudates of pepper played a key role, promoting positive interactions among microorganisms by providing specific chemical signals or nutrient substrates and constructing a complex, stable, and synergistic inter-root microbial network for disease resistance. This is an important mechanism for its biological defense effect.

### Synergistic disease-suppressive effect of *Streptomyces* and Chrysin

Metabolomic analysis revealed that REI treatment significantly upregulated the secretion of flavone compounds, such as Chrysin, from snap bean roots (Fig. 6C and D). This response builds upon the activation of upstream Shikimate and Phenylpropanoid pathways triggered by pathogen infection (36), pepper exudates may channel phenylpropanoid flux toward flavonoid synthesis by enhancing chalcone synthase activity (37). Chrysin indicated optimal disease control at 1,000 µmol·L^−1^, while higher concentrations (2,000 µmol·L^−1^) probably resulted in phytotoxicity and potentially disrupted cell membrane integrity. This dose-dependent effect is consistent with the role of flavonoids in regulating plant defense responses through MAPK signaling pathways (38, 39). This study reveals that pepper root exudates may recruit antagonistic bacteria, such as *Streptomyces,* to form a biobarrier against *F. solani* infestation through albicans, consistent with the “cry for help” theoretical framework (40). This complements mechanisms, including Arabidopsis secreting glucosinolates, to recruit antagonists (41) or soybean flavonoids regulating rhizobial interactions (42).

While this study identified the core roles of *Streptomyces* and Chrysin under controlled conditions, several limitations and future directions must be acknowledged. First, the functions of other substantially altered genera (e.g., *Pseudomonas*, *Trichoderma*) within the microbial community are not fully elucidated, necessitating future metagenomic or multi-omics approaches to comprehensively clarify microbe–metabolite interactions (43). Second, and crucial for practical application, our pot-based findings require validation under field conditions. The stability and efficacy of pepper root exudates are likely influenced by diverse soil types, climatic conditions, and agronomic practices. Furthermore, the optimal dosage for field application remains to be determined, and the specific molecular targets of Chrysin, including its potential role in modulating plant hormone pathways, such as salicylic acid or jasmonic acid, warrant further investigation. Despite these limitations, our findings illuminate promising application strategies. The precise modulation of root exudate composition, for example, through the addition of flavonoid precursors or the application of synthetic analogs like Chrysin analogs, could be employed to strategically enhance crop disease resistance. Similarly, developing *Streptomyces* inoculants using advanced formulations (e.g., nanocarrier technology) could improve their rhizosphere colonization and functional stability. Therefore, this study not only deciphers key mechanisms but also provides a tangible foundation for developing novel biological strategies to reduce dependence on chemical pesticides and advance sustainable agriculture.

### Conclusion

**Fig 8:**
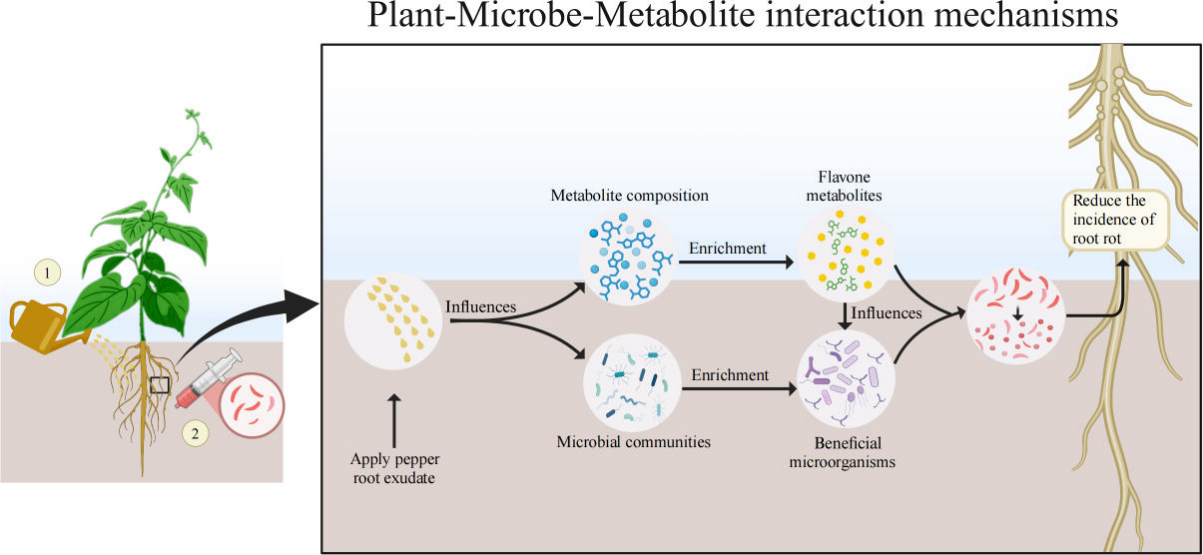
Mechanisms of plant–microbe–metabolite interactions in exogenously applied pepper root exudates.

This study reveals a dual mechanism through which the exogenous application of pepper root exudates enhances snap bean resistance to root rot: (i) reshaping the structure of the rhizosphere microbial community, particularly enriching beneficial microbes, including Actinobacteria, Proteobacteria, Ascomycota, *Saccharothrix*, *Microascus*, and *Streptomyces*; (ii) inducing the synthesis of endogenous flavone metabolites, such as Chrysin, in the recipient plant, thereby establishing a chemical–microbial synergistic defense barrier. The accumulation of Chrysin in REI demonstrated dependency on pathogen stress: synthesis was inhibited without *F. solani* infection, yet significantly activated upon *F. solani* infection. This finding not only uncovers a novel mechanism of interspecific plant signaling regulation within rhizosphere immunity (“plant–plant” signaling) but also establishes a theoretical foundation for crop green control technologies based on the modulation of root exudates (Fig. 8).

## Data Availability

Relevant data supporting the critical findings of this study are available within the article. 16S amplicon sequences were deposited in NCBI under BioProject accession number PRJNA1235524.

## SUPPLEMENTAL MATERIAL

Fig. S1 aem.01664-25-s0001.tif Neutral community model (NCM) for bacterial and fungal communities.

Fig. S2 aem.01664-25-s0002.tiff Random forest screening of key OTUs (top 50).

Table S1 aem.01664-25-s0003.xlsx Bacterial-fungal cross-domain co-occurrence network topological relationship data.

Table S2 aem.01664-25-s0004.xlsx Strain identification and NCBI accession numbers.

ASM does not own the copyrights to Supplemental Material that may be linked to, or accessed through, an article. The authors have granted ASM a non-exclusive, world-wide license to publish the Supplemental Material files. Please contact the corresponding author directly for reuse.
