# Supplementary figures and images for: Pepper root exudate attenuates snap bean root rot by mediating microbial community remodeling
Source: Appl Environ Microbiol. 2025 Oct 31;91(11):e01664-25. doi: 10.1128/aem.01664-25 (PMC12628808; doi:10.1128/aem.01664-25)

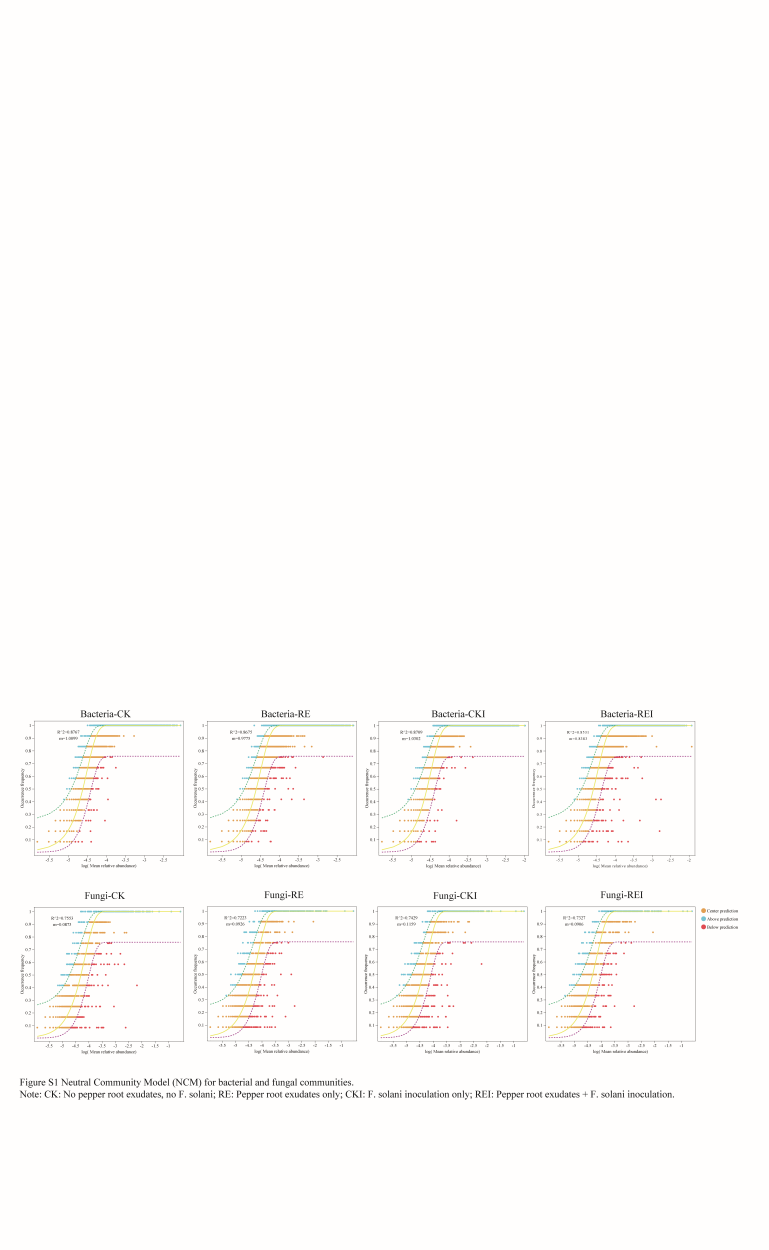

Supplement: Fig. S1 — Neutral community model (NCM) for bacterial and fungal communities. [file aem.01664-25-s0001.tif]

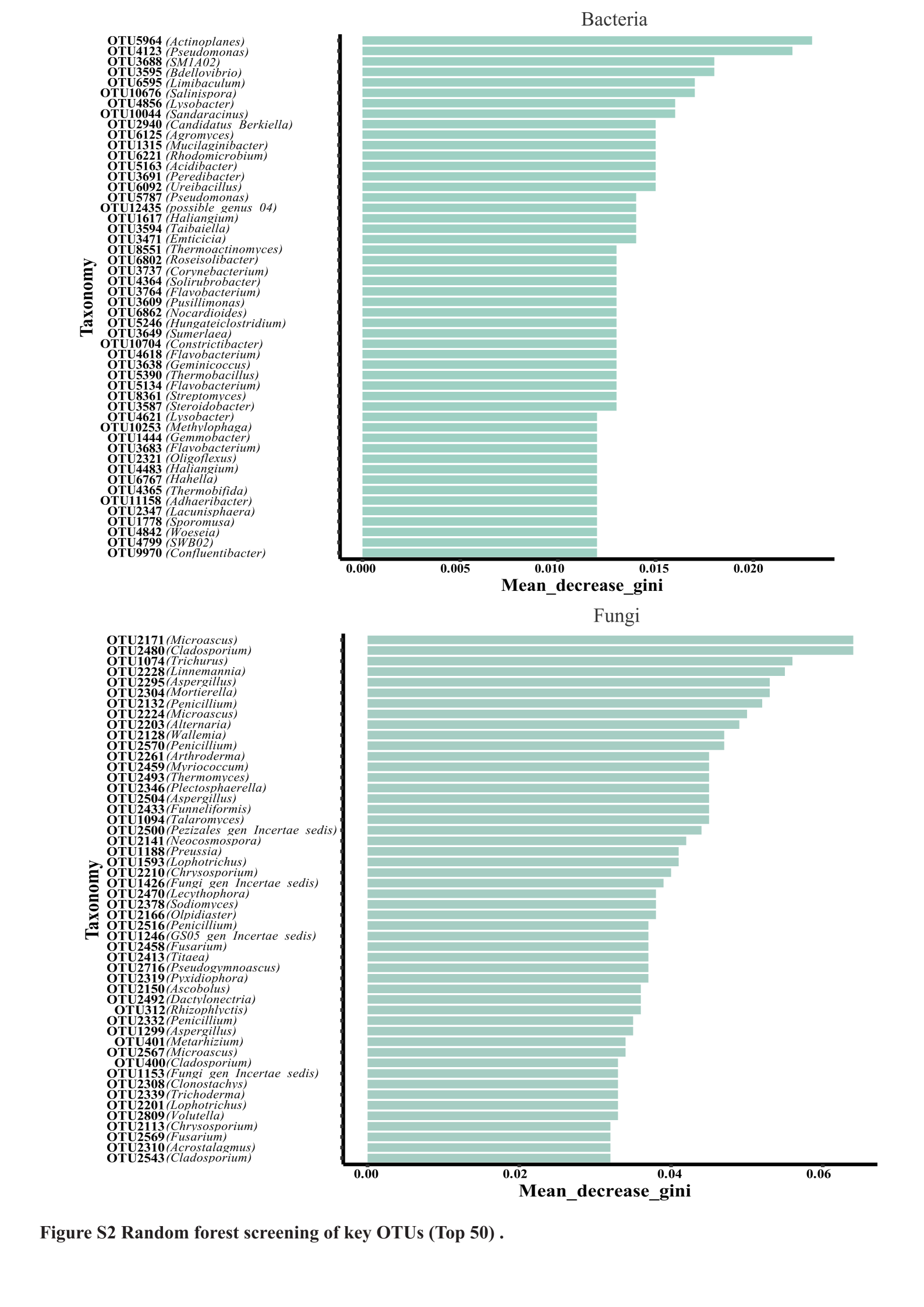

Supplement: Fig. S2 — Random forest screening of key OTUs (top 50). [file aem.01664-25-s0002.tiff]
